# Multiplex restriction amplicon sequencing: a novel next‐generation sequencing‐based marker platform for high‐throughput genotyping

**DOI:** 10.1111/pbi.13192

**Published:** 2019-07-23

**Authors:** Amy Bernardo, Paul St. Amand, Ha Quang Le, Zhenqi Su, Guihua Bai

**Affiliations:** ^1^ Department of Plant Pathology Kansas State University Manhattan KS USA; ^2^ Hard Winter Wheat Genetics Research Unit USDA‐ARS Manhattan KS USA; ^3^ Department of Agronomy Kansas State University Manhattan KS USA; ^4^ China Agricultural University Beijing China

**Keywords:** Wheat, *Triticum aestivum* L., high‐throughput genotyping, single nucleotide polymorphism, next‐generation sequencing

## Abstract

To enable rapid selection of traits in marker‐assisted breeding, markers must be technically simple, low‐cost, high‐throughput and randomly distributed in a genome. We developed such a technology, designated as Multiplex Restriction Amplicon Sequencing (MRASeq), which reduces genome complexity by polymerase chain reaction (PCR) amplification of amplicons flanked by restriction sites. The first PCR primers contain restriction site sequences at 3’‐ends, preceded by 6‐10 bases of specific or degenerate nucleotide sequences and then by a unique M13‐tail sequence which serves as a binding site for a second PCR that adds sequencing primers and barcodes to allow sample multiplexing for sequencing. The sequences of restriction sites and adjacent nucleotides can be altered to suit different species. Physical mapping of MRASeq SNPs from a biparental population of allohexaploid wheat (*Triticum aestivum* L.) showed a random distribution of SNPs across the genome. MRASeq generated thousands of SNPs from a wheat biparental population and natural populations of wheat and barley (*Hordeum vulgare L*.). This novel, next‐generation sequencing‐based genotyping platform can be used for linkage mapping to screen quantitative trait loci (QTL), background selection in breeding and many other genetics and breeding applications of various species.

## Introduction

The rapid development of molecular marker technologies has accelerated the evolution of modern plant breeding. Traditionally, breeders could only select a few traits that were visually identifiable and were usually limited to one trait per experiment (Collard and Mackill, 2007). Using marker‐assisted breeding (MAB), however, breeders can indirectly select multiple traits simultaneously including those that are not easily discerned visually (Jiang, 2013). MAB is nondestructive and only needs DNA from a small piece of tissue but can aid selection for as many traits as desired. Therefore, MAB can significantly reduce breeding cycles and improve selection efficiency (Randhawa *et al*., 2009; Yang *et al*., 2015). Previously, most plant MAB studies aimed at the integration of a few traits into an adapted background (foreground selection) using a single or a few markers for target trait selection (Jiang, 2013). Simple sequence repeats (SSRs), single nucleotide polymorphism (SNP) and sequence‐tagged site (STS) markers have been routinely used for such a purpose (Francia *et al*., 2005; Gupta *et al*., 2010). As the demand for higher crop yields and better quality rapidly increases to meet the needs of a growing world population, modern breeders are adding more traits to their selection targets (Sorrells, 2007). Thus, the list of markers linked to different quantitative trait loci (QTL) that need to be assayed during selection is quickly expanding. Furthermore, to quickly remove undesirable nontarget genome regions of donor parents in gene pyramiding experiments, marker‐assisted background selection (MABS) has been proposed to reduce selection cycles (Randhawa *et al*., 2009). In MABS, a selected set of lines, carrying the desired gene(s) in a segregating population, is genotyped with genome‐wide markers to determine the proportion of donor parent genome remaining in those lines (Semagn *et al*., 2006a). Lines carrying the target genes or QTLs and the smallest portion of the donor genome are selected for further testing. Randhawa *et al*. (2009) reported that the use of 100 SSRs resulted in 97.2% recurrent parent genome recovery at BC_2_F_2:3_. Kumar *et al*. (2010) used 61 SSR markers for background selection in three successive backcross generations to transfer a preharvest sprouting tolerance gene into bread wheat and recovered 83.6–93.4% of the recurrent parent genome. However, the high per sample cost for singleplex SSR markers prevents their routine application in breeding.

Furthermore, most agronomically important traits are controlled by many QTLs, each contributing only a small portion of the genetic variation of a trait (Jiang, 2013). Simultaneously selecting for multiple traits is becoming routine in developing modern cultivars (Sorrells, 2007). Genomic selection (GS) has recently become a popular tool that uses markers to select for multiple QTLs (Heffner *et al*., 2011). GS has been successfully used for selecting grain yield, days to heading, thousand kernel weight and grain quality traits in wheat (Heffner *et al*., 2011; Poland *et al*., 2012a). Both genome‐wide background selection and GS require the analysis of a large number of genome‐wide markers (Meuwissen *et al*., 2001; Xu *et al*., 2017). Therefore, a simple, inexpensive, high‐throughput marker platform is urgently needed for MABS, QTL mapping and GS in modern breeding.

SNPs are codominant, unlimited in number and randomly distributed in genomes (Jiang, 2013), and thus ideal for QTL mapping, MABS and GS. Several high‐throughput SNP genotyping platforms have been adopted in wheat research. Among them, high‐density SNP arrays (9K, 35K, 90K, 660K and 820K) have been developed (Cabral *et al*., 2014; Wang *et al*., 2014; https://wheat.pw.usda.gov/ggpages/topics/Wheat660_SNP_array_developed_by_CAAS.pdf; Winfield *et al*., 2016; Allen *et al*., 2017) and used for QTL and association mapping of stripe rust, plant height, grain yield and preharvest sprouting in wheat (Cabral *et al*., 2014; Sela *et al*., 2014; Sukumaran *et al*., 2015; Zhang *et al*., 2017). Recently, the 660K SNP array has been used for background selection of near‐isogenic lines carrying a powdery mildew resistance gene (Xu *et al*., 2017). However, SNP array genotyping is technically demanding, labour‐intensive, and very expensive (US$35‐250 per sample) (Bassi *et al*., 2016), and thus impractical for MABS or other routine breeding applications.

Recent advances in NGS technologies have significantly reduced genotyping costs and made it possible to use high‐throughput SNP genotyping in routine breeding (Yang *et al*., 2015). Genotyping by sequencing (GBS) has become a popular platform for breeding because it can simultaneously discover and genotype thousands of SNPs in a population (Elshire *et al*., 2011). GBS uses restriction digestion to reduce genome complexity and barcoded‐primer ligation to facilitate multiplexing samples for NGS (Elshire *et al*., 2011). In wheat, GBS has been successfully used to map preharvest sprouting QTLs (Lin *et al*., 2015), a Hessian fly resistance gene (Li *et al*., 2015), and predict breeding values through GS (Poland *et al*., 2012a). However, GBS is a proprietary technology that requires high licence and future royalty fees when using the technology to develop commercial cultivars. Also, it requires high‐quality DNA and normalized DNA concentrations for library construction (Davey *et al*., 2011).

To bridge array‐based and sequence‐based genotyping methods, Burridge *et al*. (2018) conducted targeted genotyping by sequencing (TGbyS) using SNP‐based capture probes to acquire the same sequences that hybridize to wheat genotyping arrays followed by next‐generation sequencing (NGS). TGbyS requires prior knowledge of sequence information for probe design. In polyploids such as wheat, designing genome‐specific probes is challenging, or impossible, for many targets. Furthermore, germplasms with new, rare or unique sequence variations in target regions will not be captured by predetermined probe sequences. Hence, a simple, low‐cost, high‐throughput SNP genotyping platform is urgently needed for breeding. In this study, we report such a platform for MABS and other breeding and genetic applications.

## Results

### *In silico* amplicon targets

Using *in silico* analysis, we searched for all 60 to 250 bp genomic regions flanked by *PstI* and *MspI* restriction sites (length excluded restriction sites) (Table S1) and identified 1.96 million potential target sites in the International Wheat Genome Sequencing Consortium (IWGSC) reference genome (IWGSC, 2018). Those length constraints were chosen because they are PCR amplifiable fragments, fall within NGS optimum sizes and are of the correct size for analysis by TASSEL. As expected, genome ‘B’ has the most sites (0.72 mill), with the fewest sites on the ‘D’ genome (0.55 mill).

**Figure 1:**
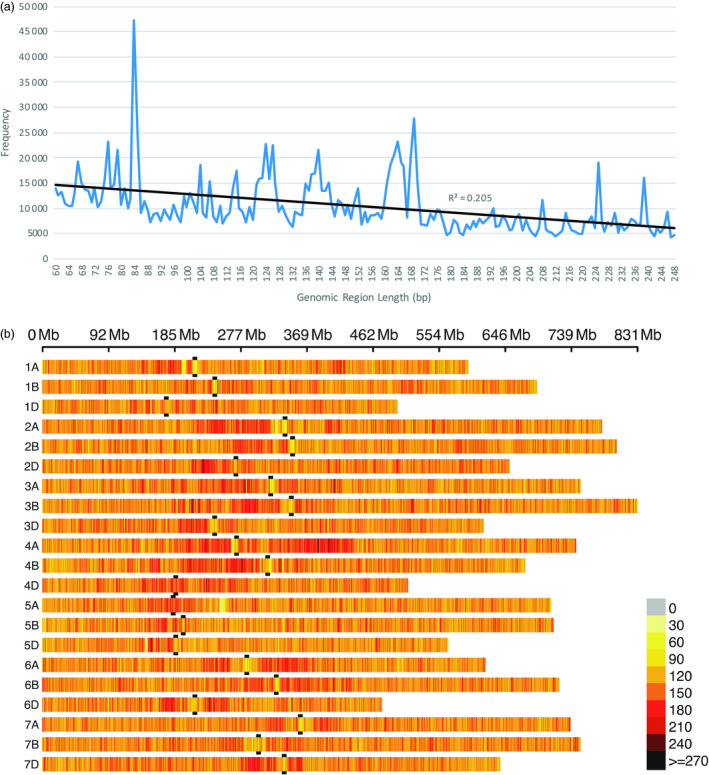
*In silico* wheat genome‐wide amplicon length frequency and distribution. Genomic regions flanked by *PstI* and *MspI* restriction enzyme sites that are from 60 to 250 bp long (excluding restriction sites) over all 21 wheat chromosomes of the IWGSC reference genome v1.0 (IWGSC, 2018). (a) Frequency and regression of *in silico* amplicon length. (b) *In silico* amplicon density distribution per 1 Mb. Black bars flank the centromere position of each chromosome.

*In silico* analysis showed that the average distance between amplicons is about 7 kb and is highly consistent across all chromosomes and among genomes (Table S2). The standard deviation is consistently small. Even the maximum gap between amplicons is relatively uniform and small, ranging from 148 to 363 kb. Within our amplicon size constraints, the frequency of *in silico* amplicon lengths shows a small bias towards shorter amplicons (r^2^ = 0.205, Figure 1a), but has a fairly uniform distribution of amplicon sizes, punctuated by several large spikes. The *in silico* distribution of amplicons per 1 Mb across the IWGSC reference genome shows that nearly all wheat chromosomes have far more amplicons nearer the centromere than occur on the distal ends of a chromosome (Figure 1b).

In an attempt to determine potential primer sequences for amplicon targets, we generated *in silico* amplicon counts for all specific 12‐mer primer pairs or 16‐mer primer pairs across the IWGSC reference genome using more restrictive amplicon length constraints (77–202 bp long, excluding restriction sites) along with primer Tm constraints. Amplicons were counted only if both primers had nearest neighbour Tms of ≥ 38 °C for 12‐mers and ≥ 50 °C for 16‐mers (Figure S1). We discovered that very few sets of specific primers are needed to produce large numbers of amplicons, indicating that a small number of primer pairs are colocated near each other many times in the wheat genome. Given these Tm and length constraints, *in silico* analysis found a total of 402 619 12‐mer primer pairs which can produce 1 237 218 total amplicons (Table S3). The best specific pair of 12‐mer primers produces 2.5% of all amplicons (30,645). The top 10 12‐mer primer pairs produce > 10% of all amplicons (126,914), the top 25 12‐mer primer pairs produce more than 200 000 amplicons, and the top 100 12‐mer primer pairs produce more than 25% of all amplicons, whereas the worst 20% of 12‐mer primer pairs produce only one amplicon each. As expected, amplicon counts were slightly lower for 16‐mer primers. *In silico* analysis found a total of 469,552 16‐mer primer pairs which produce 1 029 830 total amplicons (Table S4). The best single specific pair of 16‐mer primers produces nearly 1% of all amplicons (8915), the top 10 16‐mer primer pairs produce nearly 6% of all amplicons (61 205), the top 25 16‐mer primer pairs produce more than 100 000 amplicons, and the top 441 16‐mer primer pairs produce more than 25% of all amplicons. Fifteen per cent of 16‐mer primer pairs produce only one amplicon. Since the top 25 16‐mer or 12‐mer primer pairs produce at least 100 000 amplicons *in silico*, they were chosen for specific multiplex restriction amplicon sequencing (sMRASeq) primer testing (Tables S5 and S6).

### SNP counts using specific and degenerate primers

**Figure 2:**
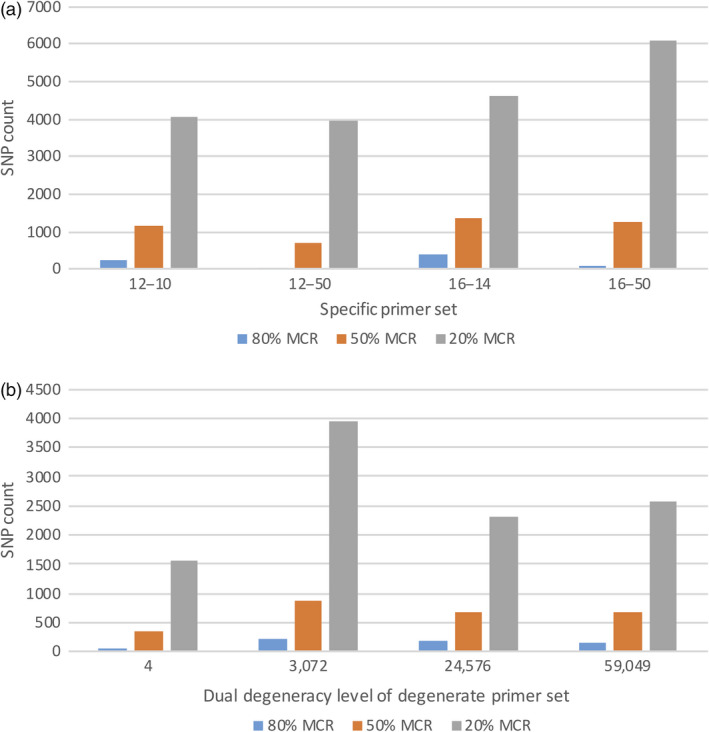
SNP counts in the Lx99 x Danby subpopulation using specific (a) and degenerate (b) primer combinations in MRASeq. (a) SNP count (Y‐axis) at 80%, 50% and 20% minimum call rate (MCR) as affected by pools of 12‐mer and 16‐mer primers (X‐axis). In the primer set name, the first two digits denote length of the *PstI* primer and the two digits after the dash line refer to the number of primers in the primer pool. Primer pairs were identified as producing the most amplicons in an *in silico* analysis of the IWGSC reference genome v1.0 (IWGSC, 2018). (b) SNP counts at 80%, 50% and 20% minimum call rate (MCR; Y‐axis) generated by different degenerate *PstI* and *MspI* primer pairs with combined degeneracy levels of 4, 3,072, 24,576 and 59,049 (X‐axis). Degenerate primer pairs were identified as producing the most amplicons in an *in silico* analysis of the IWGSC reference genome v1.0 (IWGSC,2018).

Two parents, Lx99 and Danby, and their 44 randomly selected RILs were initially analysed for polymorphic SNPs using pools of different sequence‐specific 12‐mer or 16‐mer primers (Figure 2a) that were identified as producing the most amplicons in the *in silico* analysis. The sMRASeq 16–50 primer pool contained 25 pairs of specific 16‐mer primers and generated the most SNPs (6,076) at a 20% minimum call rate (MCR), indicating that at least 20% of the samples carry each SNP. The 16–14 primer pool (a subset of the 16–50 pool) contained seven specific pairs of 16‐mer primers (Tables S5 and S6) and generated the most SNPs at the 80% MCR (369) and at the 50% MCR (1,374). In general, subset primer pools generated more SNPs than larger pools at the higher coverage criteria, and the 16‐mer primer sets produced more SNPs than the 12‐mer sets at all coverage criteria.

For degenerate multiplex restriction amplicon sequencing (dMRASeq), we evaluated primer sets with various degeneracy levels (Table S5) in the same 44 NILs to identify the best sets. We designed degenerate primers using two different approaches: 1) selecting the best primer combinations based on *in silico* analysis and 2) randomly designed primer sets at a wide range of degeneracy levels. We found that the dMRASeq primer set D2 with a degeneracy level of 3072 performed best at all MCR levels (Figure 2b) among the primer sets based on the *in silico* analysis. Among the randomly designed primer sets, we initially tested primer sets with degeneracy levels from 13 824 to 124 416 and found that primer set 48b*PstI‐*384*MspI,* with a degeneracy level of 18 432, produced the most SNPs and had the lowest amount of missing data (data not shown). Between the two types of the dMRASeq primers, primer set D2 amplified 13% more SNPs at 50% MCR and 24% more at 80% MCR compared to the randomly designed primer set 48b*PstI‐*384*MspI* (data not shown). Therefore, primer set D2 was the best among all dMRASeq primers tested. However, the number of SNPs generated by degenerate D2 primer set was 14–42% lower (depending on the MCR) than the best sequence‐specific sMRASeq primer pool (set 16–14). Thus, the sequence‐specific 16–14 primer pool was selected as the representative primer set for further characterization of MRASeq.

### Physical mapping using sMRASeq markers

**Figure 3:**
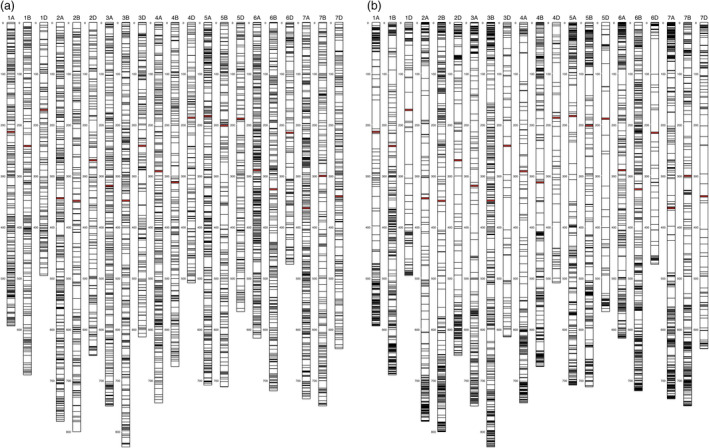
Physical maps showing the random distribution of 7,595 sMRASeq SNP markers at 20% MCR (a)and the nonrandom distribution of 7,564 GBS markers at 28% MCR with a minimum allele frequency of 0.2 or more (b)in the ‘Lx99 x Danby’ recombinant inbred wheat population. The physical location of each marker was determined by alignment to the IWGSC reference genome v1.0. Each vertical bar represents a chromosome with the chromosome number on top and megabase distance on the left. Each horizontal bar on a chromosome represents a SNP marker from one Proton sequencing run of a *PstI*‐*MspI* sMRASeq (a) or GBS (b) library. The red horizontal bars represent centromere positions.

The polymorphic SNP count from one sequencing run (typically ~80 million reads) of an sMRASeq library constructed from the full set of 184 RILs of the ‘Lx99 x Danby’ population using the 16–14 sequence‐specific primer set was 7743 SNPs at 20% MCR. About 98% of them (7595) were physically mapped to the 21 known chromosomes of the IWGSC reference genome (Figure 3a); the remaining 2% mapped to the unassigned chromosome, which holds actual sequences from the reference genome mapping project, but those sequence locations are unknown. In general, the polymorphic SNPs were randomly distributed across the 21 wheat chromosomes, with some variation in numbers of SNPs among chromosomes (Figure S2). Genome ‘A’ had the most polymorphic SNPs, followed by genomes ‘B’ and ‘D’, with the most SNPs mapped on chromosome 6A (564 SNPs) and the least on 1D (202 SNPs).

### sMRASeq in natural populations

**Figure 4:**
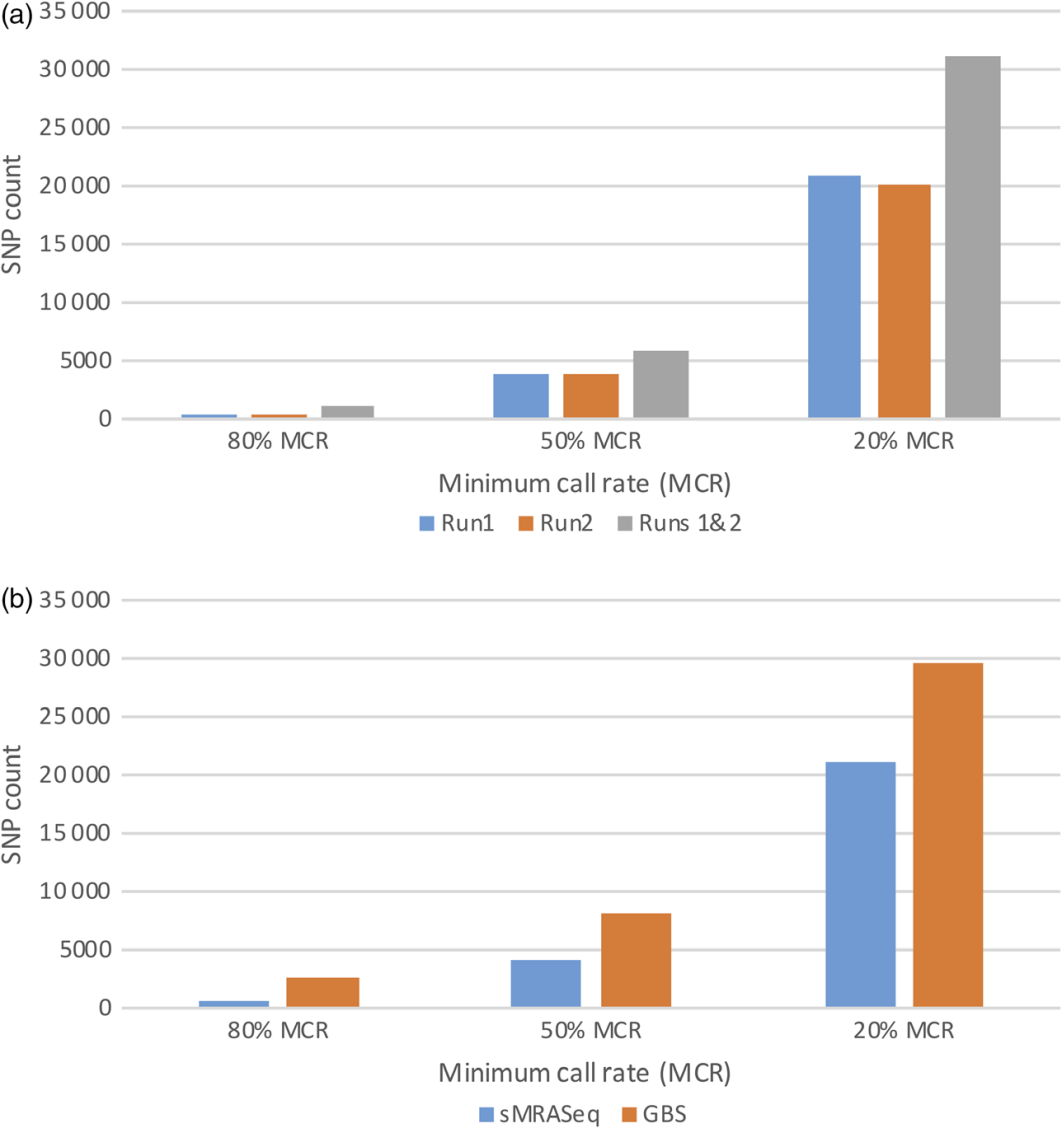
SNP counts for sMRASeq and GBS in the wheat quality association mapping population. (a) SNP counts at 80%, 50% and 20% minimum call rate (MCR; X‐axis) of sMRASeq libraries constructed using the 16–14 sequence‐specific primer set (Y‐axis). A run refers to one Proton sequencing run of the same library. (b) SNP counts (Y‐axis) at 80%, 50% and 20% minimum call rate (MCR; X‐axis) for sMRASeq using the 16–14 sequence‐specific primer set and GBS.

To evaluate the performance of sMRASeq in natural populations, a panel of 160 wheat germplasm lines were genotyped using the 16–14 sequence‐specific primer set and sequenced twice. Figure 4a shows the SNP counts from individual sMRASeq sequencing runs and the combined analysis of two sequencing runs. A single run generated an average of 537, 3968 and 20 356 polymorphic SNPs at 80%, 50% and 20% MCR, whereas the total SNPs from the two runs combined were 1108, 6012 and 31 143, respectively, indicating that additional runs significantly increased SNP counts.

The 16–14 wheat sequence‐specific sMRASeq primer set was also used to genotype a natural barley population of 96 accessions and generated 369, 1643 and 4278 SNPs at 80%, 50% and 20% MCR from one sequencing run (Figure S3), which is 1.45× to 4.82× fewer SNPs than found in wheat. However, when a pair of randomly generated dMRASeq primers (48b*PstI*‐384*MspI)* were used to genotype the barley population, the polymorphic SNP counts were 1,208, 3293 and 7467 at 80%, 50% and 20% MCR (Figure S3), which is an increase of 1.75× to 3.28× in polymorphic SNP count compared to those generated by the wheat‐specific 16–14 sMRASeq primer pool.

### sMRASeq vs GBS

sMRASeq libraries were constructed using the 16–14 primer set for 184 RILs from the ‘Lx99 x Danby’ population and a wheat association mapping population of 160 hard winter wheat accessions. GBS libraries were also constructed for the same populations.

In the wheat association mapping population, the GBS SNP count was higher than that of sMRAseq at all MCR levels (Figure 4b). SNP count differences of 4.4×, 2.0× and 1.4× were observed in favour of GBS at 80%, 50% and 20% MCR, respectively. However, the differences in SNP counts decreased as the MCR decreased. The sequencing depth of both methodologies was similar with about 26 reads per nonmissing data point.

**Figure 5:**
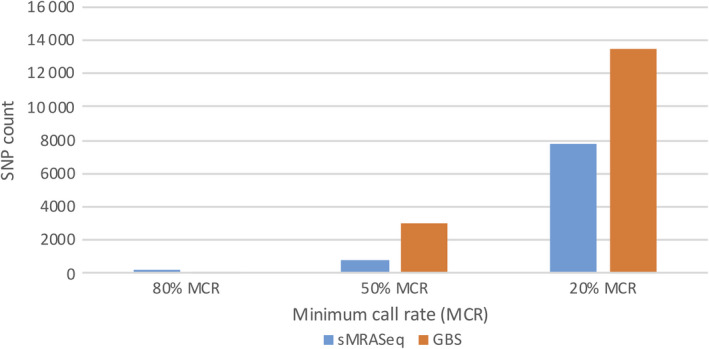
Comparison of SNP counts for sMRASeq and GBS in the ‘Lx99 x Danby’ population. SNP counts for sMRASeq using the 16–14 primer set and GBS (Y‐axis) at 20%, 50% and 80% minimum call rate (MCR; X‐axis).

In the RIL population, more SNPs were observed using sMRASeq (240) than for GBS (144) at the 80% MCR (Figure 5), but at 50% and 20% MCR, GBS generated 3.65× and 1.72× more SNPs, respectively. A physical map generated using 7595 sMRASeq SNPs at 20% MCR for the RIL population showed a relatively uniform marker distribution across all 21 wheat chromosomes (Figure 3a), whereas a GBS map of 7564 SNPs at 28% MCR (to provide similar marker numbers) showed clustered SNPs that were mostly localized in the telomeric regions of each chromosome (Figure 3b). The large marker distribution differences between these two methods indicate that sMRASeq has a much more uniform genome coverage than GBS for the same number of SNPs. The actual amplicon distribution per 1 Mb for the IWGSC reference genome v1.0 was mapped *in silico* for genomic regions flanked by *PstI* and *MspI* restriction sites that are from 60 to 250 bp long (excluding restriction sites, Figure 1). Nearly all wheat chromosomes have far more *in silico* amplicon sites nearer the centromere than occur near the telomeres. This greatly differs from the detected GBS marker distribution and agrees with the detected sMRASeq marker distribution.

## Discussion

### MRASeq is a novel, technically simple, NGS‐based marker platform

**Figure 6:**
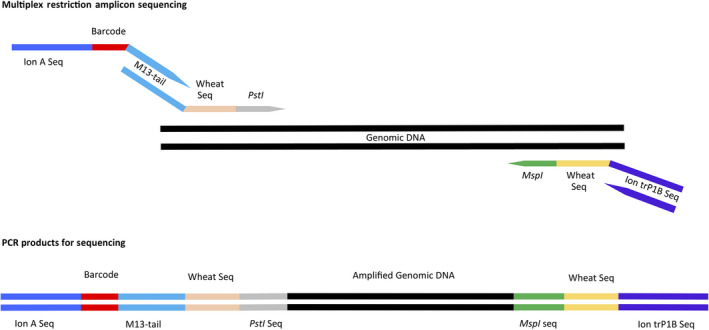
MRASeq two‐step PCR. The first PCR uses single‐stranded fusion primers with a restriction enzyme sequence on the 3’‐end of a specific or degenerate wheat sequence and an M13‐tail (forward primer) or Ion trP1B sequence (reverse primer) on the 5’‐end to amplify genomic DNA. The second PCR prepares amplicons for next‐generation sequencing using a sequencer‐specific forward primer (Ion‐A) with barcode and a sequencer‐specific reverse primer (trP1B).

Genome‐wide marker‐assisted breeding can improve and accelerate the breeding process. To be useful in breeding programmes, DNA markers must be low cost per sample, randomly distributed throughout the genome, high throughput and technically simple (Jiang, 2013). The MRASeq technique we developed is a novel NGS‐based genotyping platform that meets all those requirements. Technically, construction of an MRASeq library requires only two PCR steps (Figure 6). Through PCR primer design, we incorporate a unique M13 sequence to multiplex all amplicons in each sample and a set of barcode sequences to multiplex all samples. The first PCR of MRASeq uses restriction site‐specific primers on the 3’‐end (*PstI* on the forward primer and *MspI* on the reverse primer) and flanks the fragments to be amplified, preceded by 6–12 bases of either sequence‐specific (sMRASeq) or degenerate (dMRASeq) sequences and then an M13‐tail sequence for the forward primer or the Ion trP1B sequence for the reverse primer on the 5’‐end. The 3’‐end sequences of the forward and reverse primers bind to the *PstI* and *MspI* sequences, respectively. The sequences adjacent to restriction sites that were selected from either the *in silico* analysis of the IWGSC reference genome, or randomly designed degenerate primers, allow the primers to anneal to sequences preceding the *PstI* or *MspI* restriction sites and therefore selectively amplify target amplicons. The amplified first PCR products have an M13‐tail and *PstI* site sequence on one end and an *MspI* site and the Ion trP1B sequence on the other end creating *PstI‐MspI* amplicons. In the second PCR, the M13‐tail sequence serves as the binding site for the forward fusion primer carrying a barcode and the Ion‐A adapter sequences and the Ion trP1B serves as the reverse primer. Any sequencing platform primers could be used in place of the Ion‐specific forward and reverse sequencing primers.

Several high‐throughput genotyping platforms are available including DArT, GBS, SNP arrays, exome capture and targeted amplicon sequencing (Bernardo *et al.*, 2015; Gasc *et al*., 2016; Poland *et al*., 2012b; Semagn *et al*., 2006b; Shirasawa *et al*., 2016; Winfield *et al*., 2016). All of these platforms, except GBS, use either arrays or specific assays that are based on *a priori* sequence information from a species, have a high setup cost and a high technical demand (Bassi *et al*., 2016; Deschamps *et al*., 2012), which make them impractical to use for routine selection tools in breeding programmes. GBS does not have those requirements and its library can be constructed in‐house, thus has been a favourable platform for genome‐wide genotyping recently (He *et al*., 2014). However, GBS has been patented (United States patent US 88155112, http://www.google.com/patents/US8815512) and few public or private facilities or universities have been licensed to use the technology (https://www.keygene.com/work-with-keygene/licensing/). In addition, use of GBS requires payment of high royalty fees for variety development, which diminishes the favourability of GBS as a routine marker system for breeding applications. Technically, enzyme digestion and adapter ligation are two key steps in GBS library construction that require high DNA quality and uniform DNA concentration across samples (Davey *et al*., 2011), which adds the additional steps of DNA quantification and normalization, and thus additional assay time and cost to library preparation. To overcome those weaknesses, MRASeq targets the same genomic regions flanked by restriction sites as in GBS, but uses only two simple PCR steps to construct a library, and omits enzyme digestion and adapter ligation steps. Using MRASeq, we can easily generate thousands of high‐quality SNPs in a biparental population and tens of thousands of SNPs in a natural population. This is the first report on the use of two PCR steps to construct a library for a genome‐wide high‐throughput SNP assay, making MRASeq a novel, simple PCR platform for library construction without sacrificing library quality, and can be easily used in any basic molecular breeding laboratory.

### MRAseq is a low‐cost genome‐wide marker platform for routine breeding applications

Per sample cost is a critical factor for breeders to determine whether a marker system can be used for routine screening of breeding samples in their breeding programmes. The low cost of MRASeq makes it an attractive system for routine breeding. Currently, costs for array‐based platforms range from $35 to $250per sample (Bassi *et al*., 2016). A typical breeding programme will screen thousands of breeding samples annually, placing array‐based platforms far beyond the budgets of most breeding programmes. Although lower than array‐based platforms, initial per sample GBS costs are about $10 to $15; however, final GBS costs in a licensed facility can add up to more than $20 to $30 per sample due to required licensing fees. In this study, we demonstrated that the final cost of MRASeq was about $8 per sample ($1 per sample for DNA isolation, library construction, and clean up; $7 for library sequencing), which is much lower than that of GBS or array‐based platforms, and there are no royalty charges for cultivar development. Furthermore, the sequencing cost in this study was estimated based on a relatively low‐throughput NGS platform, the Ion Proton sequencer. High‐throughput Illumina sequencing platforms may provide far lower prices (<$5 per sample) for the same number of reads or many more sequencing reads for the same price per sample. As sequencing technology rapidly develops, sequencing costs will continue to fall, which drives MRASeq costs even lower and makes MRASeq affordable for all species in any breeding programme.

One major disadvantage of MRASeq is that it generates fewer SNPs than GBS. MRASeq only amplifies a subset of restriction sites targeted by GBS. However, genome‐wide wheat amplicon counts for specific primer pairs show that very few sets of specific primers can produce thousands of amplicons. Subsampling of all potential restriction sites is not a hindrance to finding enough markers for various applications. Both GBS and MRASeq greatly undersampled the possible number of markers of this type. For example, 4,000 markers at the 80% MRC level are only two‐tenths of a per cent of the total possible sites flanked by *PstI* and *MspI* restriction sites (Table S1). Even 30 000 markers at the 20% MRC level are only 1.5% of all possible sites. This undersampling may also partly explain why repeated GBS or MRASeq runs fail to re‐amplify the same subset of sites, since the number of sites is so large, also leading to high levels of missing data in those genotyping platforms.

The amplicon sites targeted by MRASeq are colocated near each other many times in the wheat genome. The *in silico* distribution of amplicons across the IWGSC reference genome v1.0 (International Wheat Genome Sequencing Consortium (IWGSC), 2018) shows that wheat chromosomes have far more amplicons near centromeres than occur near telomeres (Figure 1b). These data show that targeting this type of genomic site will provide numerous, uniformly spread markers. Our results show that sMRASeq markers are more uniformly distributed across the wheat genome (Figure 3a), whereas medium coverage GBS marker distribution is biased toward the distal ends of most chromosomes (Figure 3b). The favourable marker distribution of sMRASeq over GBS implies that sMRASeq needs fewer markers for equivalent genome coverage. Furthermore, the potential number of *in silico* amplicon regions flanked by *PstI* and *MspI* restriction sites is far larger than the number of markers in the 820K wheat Axiom array, the largest current marker platform (Winfield *et al*., 2016). Therefore, MRASeq can provide sufficient quality and quantity of markers for any research or breeding applications and remain within breeders’ budgets.

### Flexibility in primer design extends MRASeq application to other species

An MRASeq library can be constructed to target specific groups of amplicons by combining a small group of primers selected by *in silico* analysis of a known genome reference. In this study, we selected the seven best pairs of primers (set 16–14) that potentially amplify a huge number of fragments in wheat based on the IWGSC reference genome v1.0 (International Wheat Genome Sequencing Consortium (IWGSC), 2018) and pooled those primers for library construction. The resulting libraries produced the largest number of SNPs among all primers tested. This method can be extended to any species that has a reference genome. However, this method needs a bioinformatician to conduct *in silico* sequence analysis of a reference genome, which may be difficult for some laboratories without bioinformatics support. In this study, we provide a script for *in silico* analysis that can be directly used for any species to extract amplicons for desired criteria. The script can be easily modified for different restriction target sites that are most frequent in those species.

An MRASeq library can also be generated using degenerate primers that are designed with or without information from an *in silico* analysis of a reference genome. When data are available from an *in silico* analysis, a set of best performing primers can be assembled to aid the design of a degenerate primer set for library construction. In those species where a reference genome is not available, favourable target restriction sites can be selected based on previously reported GBS studies on that species and degenerate primers can be designed accordingly with a wide range of degeneracy levels for initial evaluation. In this study, random degenerate primers were designed following standard efficient primer design guidelines such as avoiding runs of 3 or more of one base and avoiding secondary structure formation (Robertson and Walsh‐Weller, 1998). We also aimed for 40–60% GC content and a minimum primer length of 12nt including the restriction enzyme sequence. In our preliminary study, we started with degeneracy levels from 13 824 to 124 416 to determine the proper degeneracy levels for designing random degenerate primers. Among all primers tested, one degenerate primer combination of 48b*PstI*‐384*MspI* (degeneracy levels of 48 for *PstI* sites and 384 for *MspI* sites) performed the best and was selected as the representative random degenerate primer set in this study. For specific primers, we evaluated four combinations of primers with differing lengths, Tms, and numbers of high‐amplicon targeting pairs and found that the 16–14 sequence‐specific primer pool generated more SNPs than any degenerate primer set we designed either randomly or based on the *in silico* analysis. The 16‐mer sequence‐specific primers are longer and have a higher annealing temperature for PCR than all tested degenerate primers and therefore have better specificity. Degenerate primer sets with high levels of degeneracy may generate some nonspecific amplification of DNA fragments (Shateri Najafabadi *et al*., 2008). Because MRASeq primers are designed to bind to only a portion of all the restriction site sequences targeted by GBS, the primers only subsample available specific sites that are defined by the restriction site sequences and their adjacent sequences. MRASeq primers therefore can only amplify a subset of what can be theoretically obtained by GBS, which dictates that MRAseq will generate fewer SNPs than GBS. However, reduction of the number of amplified restriction sites in MRAseq sometimes increases the sequence depth of target sequences compared to GBS (data not shown), which may reduce missing data and increase the accuracy of called SNPs. Also, different sets of degenerate primers can be designed to amplify different subsets of SNPs in different PCR runs and the resulting PCR products can be combined in a single sequencing run. Pooling PCRs from multiple, but differing, primer sets significantly increases the number of SNPs (data not shown) without increasing sequencing cost. This is especially useful in a case where one primer set does not amplify enough SNPs.

The primers we used in this study were optimized for wheat. For MRAseq in other species, different primers need to be evaluated. This study showed that the best wheat‐derived sMRASeq primer set generated fewer SNPs in barley (Figure S3), suggesting that the amplicon production was affected by the wheat‐derived primer sequences adjacent to the restriction sites. Therefore, bioinformatic priming site analysis should be used to find the best primers for MRASeq if a reference genome (or other sequence data) is available. The reference genome should be mined for the most frequent sequence pairs adjacent to targeted restriction sites and occurring within a chosen length criteria for optimum specific or degenerate primer design. If a reference genome is not available, empirical testing of different random degenerate primers is a reasonable alternative.

The restriction site sequence used in the first PCR can be altered to suit different species of interest. Aside from *PstI‐MspI*, enzyme combinations such as *EcoRI‐BfaI* for yellow mustard, *PstI‐MseI* for blackcurrant, *ApeKI‐MspI* for cabbage and *NspI*‐*BfuCI* for upland cotton have been successfully used for GBS (Fu *et al*., 2014; Islam *et al*., 2015; Lee *et al*., 2015; Russell *et al*., 2014), indicating that they can also be used to design restriction amplicon specific PCR primers for MRASeq. The change in enzyme combination does not affect the sequence of the second PCR primers; therefore, the same set of second PCR barcoded primers can be used regardless of the choice of restriction site sequence in the first PCR. This is an economic advantage over GBS where a change in restriction enzyme(s) would necessitate the change of a whole set of adaptors. In GBS, different restriction sites or restriction site combinations are used for different crops and the choice of using a frequent, moderate and/or rare‐cutting restriction enzymes affects library complexity (Fu *et al*., 2016). We chose *PstI* and *MspI* because this restriction site combination is widely used for wheat GBS and confirmed to effectively reduce wheat genome complexity and provide a usable number and quality of SNPs for various applications (Poland *et al*., 2012b).

In wheat, the large allopolyploid genome (~17 GB) makes multiplex PCR considerably more technically challenging and more complex than in other crops that have smaller genomes. Most crops are diploid with small genomes of <1 GB such as rice (389 Mb), chickpea (738 Mb) and sorghum (818 Mb). Whereas other crops have larger genomes such as maize (2.4GB),but those crop genomes are still far smaller than the wheat genome (Arumuganathan and Earle, 1991; Kim *et al*., 2005; Varshney *et al*., 2013).Regardless of genome complexity or size, we expect that MRASeq will generate sufficient SNP numbers for any marker application in any species. MRASeq shows great promise for marker‐assisted breeding in all crop species.

### Application of MRASeq

The sequence sites amplified by MRASeq are widely and uniformly distributed throughout the wheat genome. Therefore, MRASeq can be used to construct linkage maps for QTL screening. For QTL mapping, 600 genome‐wide SNPs are usually sufficient for initial QTL screening (Boehm *et al*., 2017; Kiszonas *et al*., 2017). MRASeq can easily generate > 600 high‐quality polymorphic SNPs in a biparental population of wheat. The relatively uniform distribution of these SNPs across the genome (Figure 3a) also significantly reduces the required total number of SNPs for a genome‐wide QTL scan.

MRASeq is also suitable for MABS which uses genome‐wide markers to select against unwanted residual donor genetic background. Markers help identify lines possessing the shortest target region from the onor parent, while also maximizing the recurrent parent genetic background and lowering linkage drag from the donor. MRASeq can generate thousands of uniformly distributed SNPs across a genome and is therefore particularly well suited for such an application. Additionally, pooling together MRASeq libraries with GBMAS libraries (or any targeted specific amplicon library) for sequencing (Bernardo *et al*., 2015) will enable simultaneous foreground and background selection in one sequencing run, which will significantly reduce per sample genotyping cost and marker assay time.

MRASeq may also be used for GS after imputation of missing data. Poland *et al*. (2012a) reported that the prediction accuracy of 1829 GBS markers with imputation for wheat GS was not significantly different from that of 34 749 nonimputed markers. In our study, sMRASeq generated 20 656 and 4278 SNPs at 20% MRC from one Proton run of wheat and barley populations, respectively, and these numbers are likely enough for GS after missing data are imputed. More recently, an analytic tool called the practical haplotype graph (PHG) has been developed (Johnson *et al*., 2018). This bioinformatics pipeline was designed to create a comprehensive database using all sources of sequence information available for the species of interest and then analyse target samples using low‐depth sequencing. The genome‐wide haplotypes of samples are imputed from low‐depth sequencing data using the comprehensive haplotype database. Low cost per sample and uniform marker distribution across a genome make MRASeq an ideal low‐depth or ‘skim’ sequencing technology for such breeding applications. Additionally, one or multiple pooled wheat MRASeq libraries at 20% MCR can generate enough SNPs for GWAS which requires 20 000 or more markers depending on the level of linkage disequilibrium in the populations (Ott *et al*., 2017).Thus, this simple, inexpensive genotyping platform can be used in any breeding programme with a basic laboratory setup for routine genotyping of plant breeding samples.

## Materials and methods

### Plant materials

A random set of 44 F_5:6_ recombinant inbred lines (RILs) from a cross ‘Lx99 x Danby’ and their parents were used for optimization of the MRAseq protocols. Lx99 and Danby are white winter wheat cultivars from China and Kansas, USA, respectively. To validate the utility of the new MRASeq protocol, a full set of 184 RILs from ‘Lx99 x Danby’, a panel of 160 hard winter wheat accessions and a panel of 96 diverse barley accessions were used. The barley panel was provided by the USDA National Small Grains Collection, Aberdeen, ID, USA, and the 160 hard winter wheat accessions were randomly selected from a historic collection of regional performance nurseries in the US Great Plains.

For DNA isolation, wheat seeds were planted in plastic growing trays containing Metro‐Mix 360 growing medium (Hummert Int., Topeka, KS) and seedlings were grown in a greenhouse at 20 °C with a 12 h light/dark cycle. Leaf tissues were collected at the two‐leaf stage, and genomic DNA was extracted using a modified 2% cetyltrimethylammonium bromide (CTAB) and chloroform:isoamyl (24:1) alcohol method (Saghai‐Maroof *et al*., 1984) in which 4 mM tris (2‐carboxyethyl) phosphine (TCEP) was used in place of 2‐mercaptoethanol and supplemented with 40 μg RNase (Amresco, Solon, OH). DNA was quantified using a Quant‐iT PicoGreen dsDNA HS assay kit (ThermoFisher, Waltham, MA) on a FLUOstar Omega fluorescence plate reader (BMG LABTECH, Cary, NC) and normalized to 20 ng/μL with 10 mM Tris using a Mantis liquid handling system (Formulatrix, Bedford, MA).

### *In silico* amplicon target mining

The IWGSC reference genome v1.0 (International Wheat Genome Sequencing Consortium (IWGSC), 2018) was mined using *in silico* methods for *PstI‐MspI* delimited amplicon sites using a custom C++ language program written specifically for this purpose. The program allows the use of any reference genome, most restriction enzyme pairs, any amplicon length criteria, and also calculates the nearest neighbour Tm (Owczarzy *et al*., 2004; SantaLucia and Hicks, 2004) for all possible primers (from 12 to 18 bases long including restriction site) on each side of the target amplicon. The program is available for download (https://hwwgenotyping.ksu.edu/protocols/files/Amplicon_extractor_program_by_Ha_Le.7z).

We searched for *PstI‐MspI* delimited amplicon sites that (1) are 60–250 bases apart (excluding restriction sites), (2) have no intervening *PstI* or *MspI* sites and (3) occur in either direction. The genomic position, direction, amplicon length, primer sequences and Tms were output into text files. This data set was imported into a relational database to enable searching on multiple simultaneous criteria, allowing us to subset the data for any combination of amplicon length range, primer length, specific or degenerate primer sequences, or primer Tm.

### Primer design

The MRASeq assay consists of two PCR steps (Figure 6). The first PCR amplifies genomic regions delimited by two specific restriction sites and adds a tail sequence and a reverse sequencing primer site to each amplicon. The second PCR adds a sequencing primer site and barcode to each amplicon using the tail added in the first PCR. The MRASeq assay does not use any restriction or ligation steps and is customizable using any two specific restriction enzyme sites.

The first PCR uses a forward fusion primer consisting of, from 5’ to 3’, an M13‐tail sequence (GATGTAAAACGACGGCCAGTG), 6–10 bases (XXXXXX) of either degenerate (dMRASeq) or specific (sMRASeq) sequences, and the *PstI* restriction site sequence (CTGCAG) (Figure 6). The reverse primer is comprised of the Ion truncated P1B (trP1B) adapter sequence and 6–12 bases plus the *MspI* sequence (5’‐CCTCTCTATGGGCAGTCGGTGATXXXXXXCCGG‐3’). The second PCR uses a forward primer consisting of, from 5’ to 3’, the standard Ion‐A adapter sequence (CCATCTCATCCCTGCGTGTCTCCGACTCAG), a unique barcode of 10–12 nt (Thermo Fisher Scientific, Waltham, MA) and the M13‐tail sequence, and use the Ion trP1B sequence as the reverse primer (Figure 6).

Based on the method used to obtain the sequence (6–10 nt) preceding restriction site sequences in first PCR primers, MRASeq is classified into two types: dMRASeq and sMRASeq. In dMRASeq, the primer bases between the *PstI* sequence and M13‐tail were 6 nt, degenerate and designed on primer pairs that were either (1) identified as producing large numbers of amplicons based on the *in silico* analysis of the IWGSC reference genome v1.0 (International Wheat Genome Sequencing Consortium (IWGSC), 2018) or (2) randomly generated but empirically proven to produce the most frequent amplicons among degenerate primer sets tested (Table S6). The corresponding first PCR reverse primers contained 8nt of degenerate bases between the *MspI* sequence and trP1B (CCTCTCTATGGGCAGTCGGTGAT) and were designed on either (1) primer pairs identified in the *in silico* analysis or (2) were randomly generated (Table S6).

For sMRASeq, the 6–10 bases between the M13‐tail and the *PstI* sequence in forward primer were specific and selected from primer pairs that yielded the most frequent amplicons (Tables S3 and S4) of the *in silico* analysis of the IWGSC reference genome v1.0 (Table S6). Likewise, the 8–12 bases between the *MspI* sequence (CCGG) and trP1B were specific and selected from primer pairs of the *in silico* analysis (Tables S3 and S4). In both dMRASeq and sMRASeq, the second PCR is the same (Figure 6). The specific primers were only desalted, and the degenerate primers were HPLC‐purified. We tested nine sets of MRASeq primers at degeneracy levels from 4 to 59 049 including eight sets designed based on *in silico* analysis and one set that was randomly designed (Tables S5 and S6).

### Library construction and amplicon sequencing

For the first MRASeq PCR, a 25 μL reaction mix contained 1X non‐hot start NEB *Taq* (New England BioLabs, Ipswich, MA), with 4 mM additional MgCl_2_, 100 nM each of forward and reverse primers, and 100 ng template DNA. The touch‐up PCR was set for an initial denaturation at 95°C for 1 min followed by 30 cycles of 95 °C for 1 min, 56 °C (or lower depending on primer set) for 2 min, a temperature ramp to 68 °C at 0.2 °C/sec, 68 °C for 1 min and an additional extension step at 68 °C for 5 min. The first PCR products were diluted 50 times with distilled water for the second PCR. The second PCR volume was 10 μL with 1X non‐hot start NEB *Taq* (New England BioLabs, Ipswich, MA), 400 nm each of barcoded forward and reverse primers and 2 μL of diluted DNA template. The reactions were incubated at 95 °C for 1 min followed by 15 cycles of 95 °C for 1 min, 60 °C for 15 s and 68 °C for 1 min; plus an extra extension step at 68 °C for 3 min.

The MRASeq samples amplified by the same primer set were pooled to form a library (one library refers to one primer set across all tested germplasm lines), concentrated using a Nanosep 10K Omega Ultrafiltration membrane (Pall Corporation, Port Washington, NY) and then purified using a GenCatch PCR purification kit (Epoch Life Science, Sugar Land, TX). The library was quantified using a Qubit^®^ dsDNA HS assay kit (Thermo Fisher Scientific). About 500 ng of the library was run on a 2% E‐Gel^®^ SizeSelect^™^ Gel (Thermo Fisher Scientific) to select PCR fragments from 175 to 300 bp. Size‐selected DNA fragments were purified again using a GenCatch PCR purification kit (Epoch Life Science) and quantified using a Qubit^®^ dsDNA HS assay kit (Thermo Fisher Scientific). Equimolar concentrations of libraries (25 pM) were pooled, loaded into an Ion Chef instrument using a Hi‐Q Chef kit for automated library and template preparations, robotically loaded into PI v3 chips and then sequenced in an Ion Torrent Proton Sequencer using a Hi‐Q sequencing kit (Thermo Fisher Scientific) and default sequencer settings for Ion Torrent Suite 5.10.

For GBS samples, libraries were constructed as described by Mascher *et al*. (2013), size selected for 200–300 bp fragments, purified, quantified and sequenced as described for MRASeq using 80 pM of each library.

### Data analysis

The Ion Torrent system produces sequence reads of variable lengths. Prior to marker discovery and mapping analysis, we appended 80 poly‐A bases to all sequencing reads on their 3’‐end so that TASSEL 5.0 would attempt to use reads shorter than 64 bases rather than discarding short reads. The TASSEL pipeline expects each read to have a barcode immediately followed by a remnant‐restriction site prior to any genomic sequence. All MRASeq reads must be pre‐processed to remove the tail and specific or degenerate sequence between the barcode and remnant‐restriction site. We used the UNIX program "sed" to search for and replace intervening sequences. TASSEL analysis of MRASeq reads is identical to GBS once the reads have been pre‐processed. Note that the corresponding quality string in the fastq must also be adjusted if quality filtering will be used. The default TASSEL pipeline does not use quality filtering, nor did we filter on read quality scores in this study.

We used the TASSEL 5.0 GBSv2 discovery pipeline (Bradbury *et al*., 2007, www.maizegenetics.net) and BWA Ver. 0.7.17 (Li and Durbin, 2009) to identify SNP markers and map all reads to the IWGSC reference genome v1.0 (International Wheat Genome Sequencing Consortium (IWGSC), 2018). A minimum allele frequency (MAF) threshold of at least 0.2 (biparental populations) or 0.05 (natural populations) and a maximum of 10% heterozygous calls in the Lx99/Danby population was used to filter out low‐quality tags. All other TASSEL 5.0 settings were defaults.

## Conflict of interest

The authors declare no competing financial interests.

## Author contributions

AB, PST and GB conceived the study. GB provided reagents and oversaw the research. ZS provided materials. AB conducted experiments. PST and AB performed data analysis. LQH programmed the custom software. AB, PST and GB wrote the manuscript, and all authors approved the final manuscript.

## Supporting information


**Figure S1** Genome‐wide wheat amplicon counts for specific primer pairs.

**Figure S2** Number of sMRASeq polymorphic SNPs in the ‘Lx99 x Danby’ RIL wheat population.

**Figure S3** SNP counts in a natural barley population using MRASeq.

**Table S1** Genome‐wide amplicon counts. *In silico* amplicon counts across all 21 wheat chromosomes for genomic regions flanked by *PstI* and *MspI* restriction sites that are from 60 to 250 bp long (excluding restriction sites) from the IWGSC reference genome.

**Table S2** Distance between amplicons.

**Table S3** Number of amplicons produced by specific 12‐mer primer pairs.

**Table S4** Number of amplicons produced by specific 16‐mer primer pairs.

**Table S5** MRASeq primer set information.

**Table S6** Sequence of MRASeq primers.

## Data Availability

The *in silico* amplicon target mining programme is available for download at https://hwwgenotyping.ksu.edu/protocols/files/Amplicon_extractor_program_by_Ha_Le.7z
