# Supplementary material for: Multiplex restriction amplicon sequencing: a novel next‐generation sequencing‐based marker platform for high‐throughput genotyping
Source: Plant Biotechnol J. 2019 Jul 23;18(1):254–65. doi: 10.1111/pbi.13192 (PMC6920337; doi:10.1111/pbi.13192)
Supplement: Supplementary file 1 — Figure S1 Genome‐wide wheat amplicon counts for specific primer pairs. [file PBI-18-254-s006.pdf]

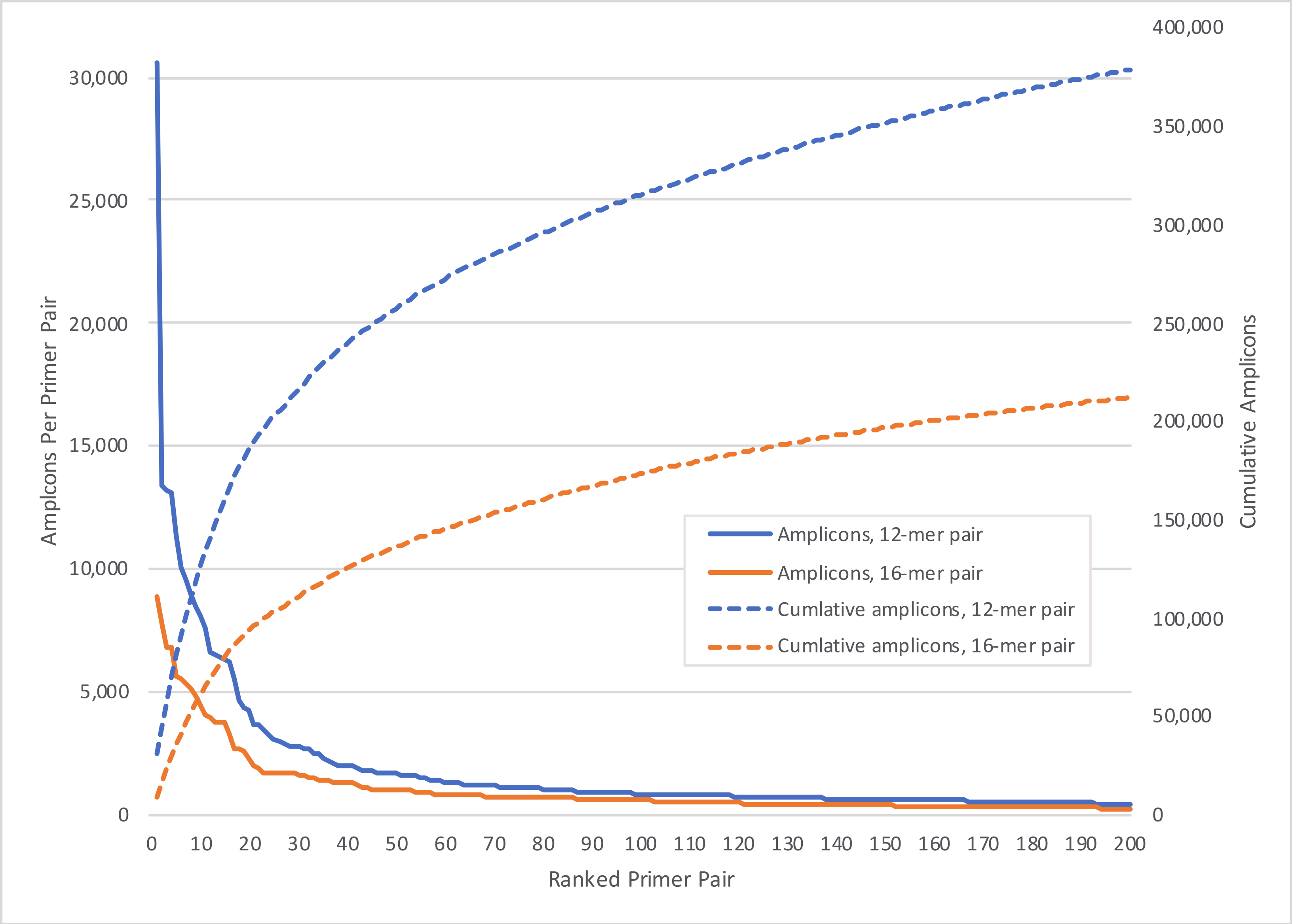

Figure S1. Genome-wide wheat amplicon counts for specific primer pairs.
In silico amplicon counts and cumulative amplicon counts for specific 12-mer primer pairs or specific 16-mer primer pairs across all 21 wheat chromosomes for genomic regions flanked by *PstI* and *MspI* restriction enzyme sites that are from 60 to 250 bp long (excluding restriction sites) based on the IWGSC reference genome v1.0 (IWGSC, 2018). Only the top ranking 200 primer pairs for each primer length shown. Amplicons were counted only if both primers had nearest neighbor Tms of ≥ 38°C for 12-mers and ≥ 50°C for 16-mers.
