# Supplementary material for: Multiplex restriction amplicon sequencing: a novel next‐generation sequencing‐based marker platform for high‐throughput genotyping
Source: Plant Biotechnol J. 2019 Jul 23;18(1):254–65. doi: 10.1111/pbi.13192 (PMC6920337; doi:10.1111/pbi.13192)
Supplement: Supplementary file 2 — Figure S2 Number of sMRASeq polymorphic SNPs in the ‘Lx99 x Danby’ RIL wheat population. [file PBI-18-254-s004.pdf]

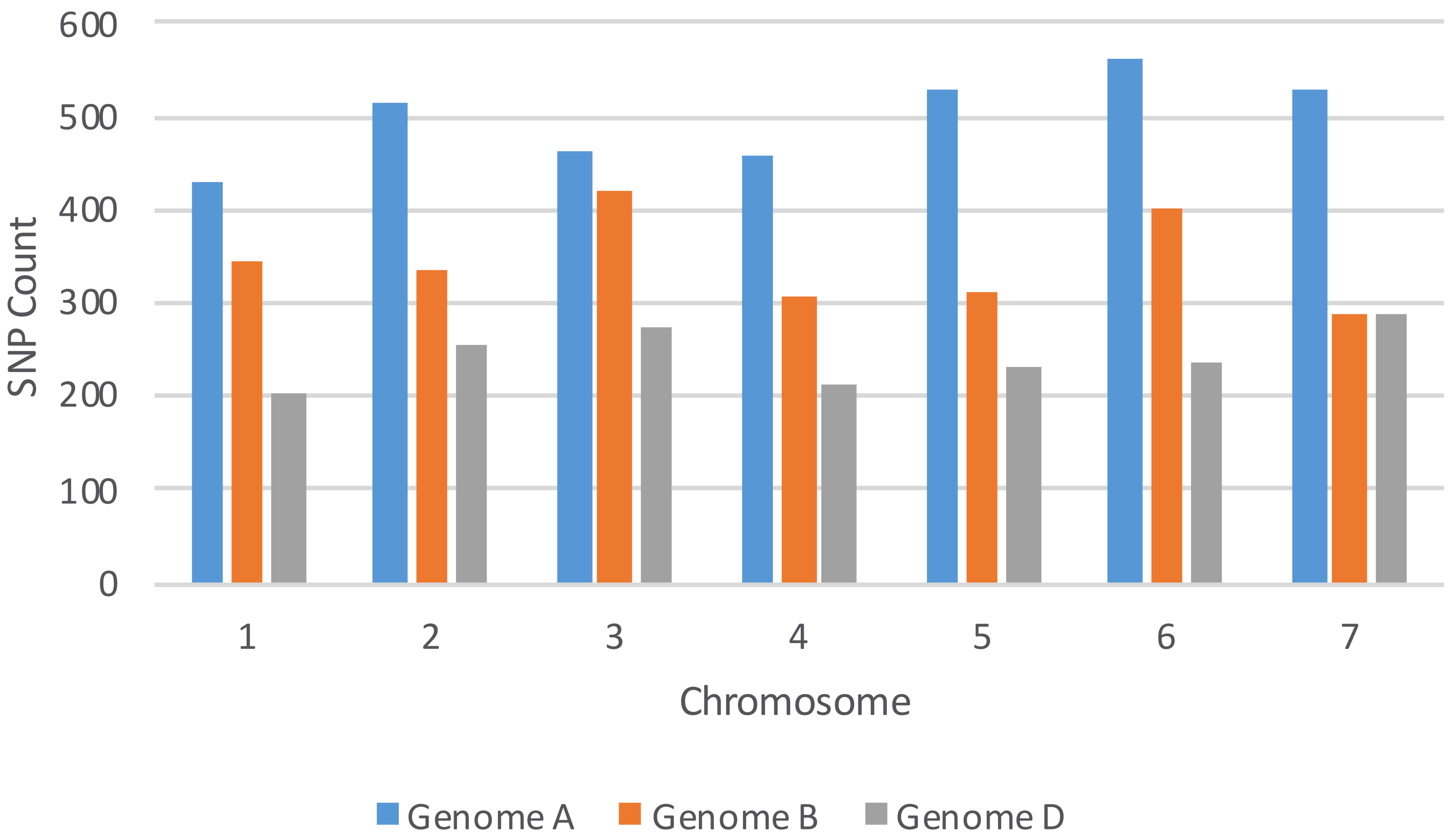

Figure S2. Number of sMRASeq polymorphic SNPs in the 'Lx99 x Danby' RIL wheat population. The number of SNPs at the 20% MCR in the 'A', 'B' and 'D' genomes of the seven wheat chromosomes from one Proton sequencing run of an sMRASeq library constructed using the 16-14 sequence-specific primer set.
