# Supplementary material for: Multiplex restriction amplicon sequencing: a novel next‐generation sequencing‐based marker platform for high‐throughput genotyping
Source: Plant Biotechnol J. 2019 Jul 23;18(1):254–65. doi: 10.1111/pbi.13192 (PMC6920337; doi:10.1111/pbi.13192)
Supplement: Supplementary file 3 — Figure S3 SNP counts in a natural barley population using MRASeq. [file PBI-18-254-s008.pdf]

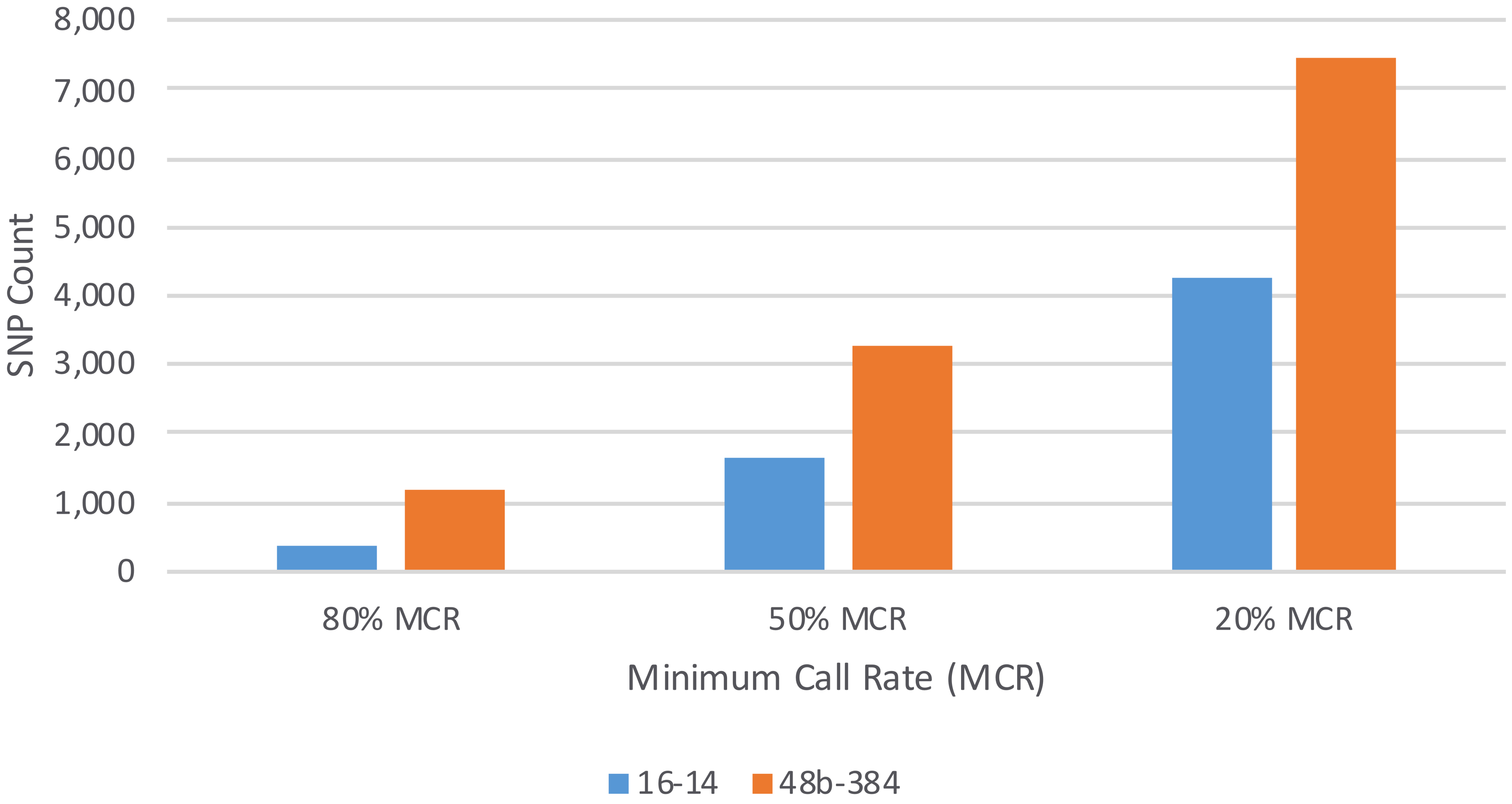

Figure S3. SNP counts in a natural barley population using MRASeq. SNP counts in barley at 80%, 50% and 20% minimum call rate (MCR) using a sMRASeq wheat specific sequence based non-degenerate 16-14 primer set and a dMRASeq random degenerate 48b*PstI*-384*MspI* primer set.
