# Supplementary material for: Multiplex restriction amplicon sequencing: a novel next‐generation sequencing‐based marker platform for high‐throughput genotyping
Source: Plant Biotechnol J. 2019 Jul 23;18(1):254–65. doi: 10.1111/pbi.13192 (PMC6920337; doi:10.1111/pbi.13192)
Supplement: Supplementary file 4 — Table S1 Genome‐wide amplicon counts. In silico amplicon counts across all 21 wheat chromosomes for genomic regions flanked by PstI and MspI restriction sites that are from 60 to 250 bp long (excluding restriction sites) from the IWGSC reference genome. [file PBI-18-254-s001.pdf]

Supplemental Table S1. In silico amplicon counts across all 21 wheat chromosomes for genomic regions flanked by *PstI* and *MspI* restriction sites that are from 60 to 250 bp long (excluding restriction sites) from the IWGSC reference genome

	Genome			
Chromosome	A	B	D	Sums
1	80,600	94,970	68,423	243,993
2	109,302	110,399	92,917	312,418
3	104,162	116,237	86,723	307,122
4	106,849	95,210	72,980	275,039
5	96,629	97,220	76,465	270,314
6	86,673	101,726	66,843	255,242
7	101,340	104,536	89,809	295,685
Sums	685,555	720,298	554,160	1,959,813
