# Supplementary material for: Multiplex restriction amplicon sequencing: a novel next‐generation sequencing‐based marker platform for high‐throughput genotyping
Source: Plant Biotechnol J. 2019 Jul 23;18(1):254–65. doi: 10.1111/pbi.13192 (PMC6920337; doi:10.1111/pbi.13192)
Supplement: Supplementary file 5 — Table S2 Distance between amplicons. [file PBI-18-254-s002.pdf]

Table S2. Distance between amplicons. In silico amplicon distance (bp) for the 21 wheat chromosomes. Distance between amplicons of genomic regions flanked by *PstI* and *MspI* restriction sites that are from 60 to 250 bp long (excluding restriction sites) from the IWGSC reference genome v1.0 (IWGSC, 2018).

		Chromosme						
Genome		1	2	3	4	5	6	7
	Maximum	163284*	182,683	147,772	222,558	175,789	162,315	149,302
	Average	7,371	7,143	7,208	6,969	7,345	7,131	7,269
A	SD	10,176	9,896	9,886	9,666	10,208	9,810	9,936
	Maximum	322,911	146,540	174,429	297,583	299,314	159,091	212,992
	Average	7,264	7,258	7,147	7,073	7,335	7,088	7,180
B	SD	10,135	10,035	9,900	9,881	10,227	9,813	9,879
	Maximum	213,601	223,155	157,594	260,173	363,415	259,845	153,045
	Average	7,241	7,030	7,097	6,985	7,403	7,084	7,111
D	SD	9,783	9,583	9,568	9,651	10,332	9,719	9,564
