# Supplementary material for: Multiplex restriction amplicon sequencing: a novel next‐generation sequencing‐based marker platform for high‐throughput genotyping
Source: Plant Biotechnol J. 2019 Jul 23;18(1):254–65. doi: 10.1111/pbi.13192 (PMC6920337; doi:10.1111/pbi.13192)
Supplement: Supplementary file 8 — Table S5 MRASeq primer set information. [file PBI-18-254-s005.pdf]

Table S5. MRASeq primer set information

Primer Set	Forward Primer(s)	Reverse Primer(s)	Degeneracy	Theoretical amplicons in Chinese Spring	1st PCR Annealing Temp, °C	MRASeq Type
12-10	M13-PstI-12.1, 12.6, 12.16, 12.21, 12.25	trP1B-MspI-12.1, 12.6, 12.16, 12.21, 12.25	10	53717	46	sMRASeq
12-50	M13-PstI-12.1 to 12.25	trP1B-MspI-12.1 to 12.25	50	202659	38	sMRASeq
16-14	M13-PstI-16.3, 16.7, 16.9, 16.11, 16.15, 16.18, 16.24	trP1B-MspI-16.3, 16.7, 16.9, 16.11, 16.15, 16.18, 16.24	14	29226	56	sMRASeq
16-50	M13-PstI-16.1 to 16.25	trP1B-MspI-16.1 to 16.25	50	103157	50	sMRASeq
D1	M13-PstI-D1	trP1B-MspI-D1	4	28645	40	dMRASeq, *in silico* -based
D2	M13-PstI-D2	trP1B-MspI-D2	3072	65590	38	dMRASeq, *in silico* -based
D3	M13-PstI-D3	trP1B-MspI-D3	24576	39530	38	dMRASeq, *in silico* -based
D4	M13-PstI-D4	trP1B-MspI-D4	59049	150045	45	dMRASeq, *in silico* -based
48bPstI-384MspI	M13-48b-PstI	trP1B-384-MspI	18432	16842	38	dMRASeq, randomly designed
