# Supplementary material for: Multiplex restriction amplicon sequencing: a novel next‐generation sequencing‐based marker platform for high‐throughput genotyping
Source: Plant Biotechnol J. 2019 Jul 23;18(1):254–65. doi: 10.1111/pbi.13192 (PMC6920337; doi:10.1111/pbi.13192)
Supplement: Supplementary file 9 — Table S6 Sequence of MRASeq primers. [file PBI-18-254-s007.zip › pbi13192-sup-0009-TableS6.pdf]

Table S6. Sequence of MRASeq primers.

Primer Name	Sequence, 5' to 3'	Purification
M13-PstI-12.1	GATGTAAAACGACGGCCAGTGGTGGAGCTGCAG	Standard
M13-PstI-12.2	GATGTAAAACGACGGCCAGTGTTAACCCTGCAG	Standard
M13-PstI-12.3	GATGTAAAACGACGGCCAGTGTTAACCCTGCAG	Standard
M13-PstI-12.4	GATGTAAAACGACGGCCAGTGTTAACCCTGCAG	Standard
M13-PstI-12.5	GATGTAAAACGACGGCCAGTGACTTTGCTGCAG	Standard
M13-PstI-12.6	GATGTAAAACGACGGCCAGTGCGTAGCCTGCAG	Standard
M13-PstI-12.7	GATGTAAAACGACGGCCAGTGACTGAACTGCAG	Standard
M13-PstI-12.8	GATGTAAAACGACGGCCAGTGACTGAACTGCAG	Standard
M13-PstI-12.9	GATGTAAAACGACGGCCAGTGTTAACCCTGCAG	Standard
M13-PstI-12.10	GATGTAAAACGACGGCCAGTGTTAACCCTGCAG	Standard
M13-PstI-12.11	GATGTAAAACGACGGCCAGTGGCATTCTGCAG	Standard
M13-PstI-12.12	GATGTAAAACGACGGCCAGTGTACACTCTGCAG	Standard
M13-PstI-12.13	GATGTAAAACGACGGCCAGTGTTGAGTCTGCAG	Standard
M13-PstI-12.14	GATGTAAAACGACGGCCAGTGATAACCCTGCAG	Standard
M13-PstI-12.15	GATGTAAAACGACGGCCAGTGACACCCCTGCAG	Standard
M13-PstI-12.16	GATGTAAAACGACGGCCAGTGGAAGCCCTGCAG	Standard
M13-PstI-12.17	GATGTAAAACGACGGCCAGTGAAATCCCTGCAG	Standard
M13-PstI-12.18	GATGTAAAACGACGGCCAGTGCTTCCACTGCAG	Standard
M13-PstI-12.19	GATGTAAAACGACGGCCAGTGATACCGCTGCAG	Standard
M13-PstI-12.20	GATGTAAAACGACGGCCAGTGTTGAGTCTGCAG	Standard
M13-PstI-12.21	GATGTAAAACGACGGCCAGTGCGAAGCCTGCAG	Standard
M13-PstI-12.22	GATGTAAAACGACGGCCAGTGATTGCACTGCAG	Standard
M13-PstI-12.23	GATGTAAAACGACGGCCAGTGCAAACTCTGCAG	Standard
M13-PstI-12.24	GATGTAAAACGACGGCCAGTGAAAACCCTGCAG	Standard
M13-PstI-12.25	GATGTAAAACGACGGCCAGTGGTGAGGCTGCAG	Standard
trP1B-MspI-12.1	CCTCTCTATGGGCAGTCGGTGATGGCGGAGTCCGG	Standard
trP1B-MspI-12.2	CCTCTCTATGGGCAGTCGGTGATGCGAAACTCCGG	Standard
trP1B-MspI-12.3	CCTCTCTATGGGCAGTCGGTGATAACTCCGGCCGG	Standard
trP1B-MspI-12.4	CCTCTCTATGGGCAGTCGGTGATAACCTCGGCCGG	Standard
trP1B-MspI-12.5	CCTCTCTATGGGCAGTCGGTGATTATGCTCCCCGG	Standard
trP1B-MspI-12.6	CCTCTCTATGGGCAGTCGGTGATGGGAGTTCCCGG	Standard
trP1B-MspI-12.7	CCTCTCTATGGGCAGTCGGTGATGGGGATCGCCGG	Standard
trP1B-MspI-12.8	CCTCTCTATGGGCAGTCGGTGATGAGGATCGCCGG	Standard
trP1B-MspI-12.9	CCTCTCTATGGGCAGTCGGTGATGCGACACCCCGG	Standard
trP1B-MspI-12.10	CCTCTCTATGGGCAGTCGGTGATCACCCCGGCCGG	Standard
trP1B-MspI-12.11	CCTCTCTATGGGCAGTCGGTGATAGGCACCTCCGG	Standard
trP1B-MspI-12.12	CCTCTCTATGGGCAGTCGGTGATTAGGTGTACCGG	Standard
trP1B-MspI-12.13	CCTCTCTATGGGCAGTCGGTGATTTATGTTCCCGG	Standard
trP1B-MspI-12.14	CCTCTCTATGGGCAGTCGGTGATAACCTCGGCCGG	Standard
trP1B-MspI-12.15	CCTCTCTATGGGCAGTCGGTGATTTCACTTCCCGG	Standard
trP1B-MspI-12.16	CCTCTCTATGGGCAGTCGGTGATCGTACAGCCCGG	Standard
trP1B-MspI-12.17	CCTCTCTATGGGCAGTCGGTGATTCTTGTAACCGG	Standard
trP1B-MspI-12.18	CCTCTCTATGGGCAGTCGGTGATGATCTCCGCCGG	Standard
trP1B-MspI-12.19	CCTCTCTATGGGCAGTCGGTGATCCATATGTCCGG	Standard
trP1B-MspI-12.20	CCTCTCTATGGGCAGTCGGTGATCCGTCATACCGG	Standard
trP1B-MspI-12.21	CCTCTCTATGGGCAGTCGGTGATTCCTCAGTCCGG	Standard

trP1B-MspI-12.22	CCTCTCTATGGGCAGTCGGTGATCACCCCCACCGG	Standard
trP1B-MspI-12.23	CCTCTCTATGGGCAGTCGGTGATCTTGTTCACCGG	Standard
trP1B-MspI-12.24	CCTCTCTATGGGCAGTCGGTGATTCTTGTAACCGG	Standard
trP1B-MspI-12.25	CCTCTCTATGGGCAGTCGGTGATGGCGGAGTCCGG	Standard
M13-PstI-16.1	GATGTAAAACGACGGCCAGTGGTGGACTGAACTGCAG	Standard
M13-PstI-16.2	GATGTAAAACGACGGCCAGTGCATGTTAACCCTGCAG	Standard
M13-PstI-16.3	GATGTAAAACGACGGCCAGTGTGAGGTGGAGCTGCAG	Standard
M13-PstI-16.4	GATGTAAAACGACGGCCAGTGTTCTGGCATTCTGCAG	Standard
M13-PstI-16.5	GATGTAAAACGACGGCCAGTGACCCTTGAGTCTGCAG	Standard
M13-PstI-16.6	GATGTAAAACGACGGCCAGTGATATGTGGAGCTGCAG	Standard
M13-PstI-16.7	GATGTAAAACGACGGCCAGTGTGGCGAAGCCCTGCAG	Standard
M13-PstI-16.8	GATGTAAAACGACGGCCAGTGTGGGACACCCCTGCAG	Standard
M13-PstI-16.9	GATGTAAAACGACGGCCAGTGAACGCGTAGCCTGCAG	Standard
M13-PstI-16.10	GATGTAAAACGACGGCCAGTGTGAGGTGGAGCTGCAG	Standard
M13-PstI-16.11	GATGTAAAACGACGGCCAGTGCTGCCTTCCACTGCAG	Standard
M13-PstI-16.12	GATGTAAAACGACGGCCAGTGGTGGACTGAACTGCAG	Standard
M13-PstI-16.13	GATGTAAAACGACGGCCAGTGACCCTTGAGTCTGCAG	Standard
M13-PstI-16.14	GATGTAAAACGACGGCCAGTGGTGGACTGAACTGCAG	Standard
M13-PstI-16.15	GATGTAAAACGACGGCCAGTGTGAGGTGGAGCTGCAG	Standard
M13-PstI-16.16	GATGTAAAACGACGGCCAGTGAAACACTTTGCTGCAG	Standard
M13-PstI-16.17	GATGTAAAACGACGGCCAGTGCATGTTAACCCTGCAG	Standard
M13-PstI-16.18	GATGTAAAACGACGGCCAGTGAGCGATTGCACTGCAG	Standard
M13-PstI-16.19	GATGTAAAACGACGGCCAGTGGTTCAAATCCCTGCAG	Standard
M13-PstI-16.20	GATGTAAAACGACGGCCAGTGTGACCGAAGCCTGCAG	Standard
M13-PstI-16.21	GATGTAAAACGACGGCCAGTGCATGTTAACCCTGCAG	Standard
M13-PstI-16.22	GATGTAAAACGACGGCCAGTGGTCCCAACCCCTGCAG	Standard
M13-PstI-16.23	GATGTAAAACGACGGCCAGTGTAGGTTAACCCTGCAG	Standard
M13-PstI-16.24	GATGTAAAACGACGGCCAGTGGCAGCTCCATCTGCAG	Standard
M13-PstI-16.25	GATGTAAAACGACGGCCAGTGGTTCAAATCCCTGCAG	Standard
trP1B-MspI-16.1	CCTCTCTATGGGCAGTCGGTGATAGGAGGGGATCGCCGG	Standard
trP1B-MspI-16.2	CCTCTCTATGGGCAGTCGGTGATGGGAAACCTCGGCCGG	Standard
trP1B-MspI-16.3	CCTCTCTATGGGCAGTCGGTGATTCTCGGCGGAGTCCGG	Standard
trP1B-MspI-16.4	CCTCTCTATGGGCAGTCGGTGATTAGTAGGCACCTCCGG	Standard
trP1B-MspI-16.5	CCTCTCTATGGGCAGTCGGTGATGGTGTTATGTTCCCGG	Standard
trP1B-MspI-16.6	CCTCTCTATGGGCAGTCGGTGATTCTCGGCGGAGTCCGG	Standard
trP1B-MspI-16.7	CCTCTCTATGGGCAGTCGGTGATCACACGTACAGCCCGG	Standard
trP1B-MspI-16.8	CCTCTCTATGGGCAGTCGGTGATCGTGTTCACTTCCCGG	Standard
trP1B-MspI-16.9	CCTCTCTATGGGCAGTCGGTGATTTCGGGGAGTTCCCGG	Standard
trP1B-MspI-16.10	CCTCTCTATGGGCAGTCGGTGATTTCCGGCGGAGTCCGG	Standard
trP1B-MspI-16.11	CCTCTCTATGGGCAGTCGGTGATTGATGATCTCCGCCGG	Standard
trP1B-MspI-16.12	CCTCTCTATGGGCAGTCGGTGATAGGAGAGGATCGCCGG	Standard
trP1B-MspI-16.13	CCTCTCTATGGGCAGTCGGTGATGTTTCCGTCATACCGG	Standard
trP1B-MspI-16.14	CCTCTCTATGGGCAGTCGGTGATAGGGGAGGATCGCCGG	Standard
trP1B-MspI-16.15	CCTCTCTATGGGCAGTCGGTGATTTTCGGCGGAGTCCGG	Standard
trP1B-MspI-16.16	CCTCTCTATGGGCAGTCGGTGATGAGCTATGCTCCCCGG	Standard
trP1B-MspI-16.17	CCTCTCTATGGGCAGTCGGTGATGCGACACCCCGGCCGG	Standard
trP1B-MspI-16.18	CCTCTCTATGGGCAGTCGGTGATCATCCACCCCCACCGG	Standard
trP1B-MspI-16.19	CCTCTCTATGGGCAGTCGGTGATTGTCTTGTAGAACCGG	Standard

trP1B-MspI-16.20	CCTCTCTATGGGCAGTCGGTGATTAGATCCTCAGTCCGG	Standard
trP1B-MspI-16.21	CCTCTCTATGGGCAGTCGGTGATGTGAGCGACACCCCGG	Standard
trP1B-MspI-16.22	CCTCTCTATGGGCAGTCGGTGATCTCCCGTCCGTGCCGG	Standard
trP1B-MspI-16.23	CCTCTCTATGGGCAGTCGGTGATGTGAGCGAAACTCCGG	Standard
trP1B-MspI-16.24	CCTCTCTATGGGCAGTCGGTGATTCCGGTGGCTAGCCGG	Standard
trP1B-MspI-16.25	CCTCTCTATGGGCAGTCGGTGATTGTCTTGTTGTACCGG	Standard
M13-PstI-D1	GATGTAAAACGACGGCCAGTGTTAACCCTGCAG	HPLC
trP1B-MspI-D1	CCTCTCTATGGGCAGTCGGTGATAACYYCGGCCGG	HPLC
M13-PstI-D2	GATGTAAAACGACGGCCAGTGKTRRMSCTGCAG	HPLC
trP1B-MspI-D2	CCTCTCTATGGGCAGTCGGTGATRVSIIMSKCCGG	HPLC
M13-PstI-D3	GATGTAAAACGACGGCCAGTGWKMRYSCTGCAG	HPLC
trP1B-MspI-D3	CCTCTCTATGGGCAGTCGGTGATWRSYYHRSCCGG	HPLC
M13-PstI-D4	GATGTAAAACGACGGCCAGTGDBIIHVCTGCAG	HPLC
trP1B-MspI-D4	CCTCTCTATGGGCAGTCGGTGATIDBIVHBVCCGG	HPLC
M13-48b-PstI	GATGTAAAACGACGGCCAGTGBRYGWSCTGCAG	HPLC
trP1B-384-MspI	CCTCTCTATGGGCAGTCGGTGATRWSWYSHWCCGG	HPLC
