# Supplementary material for: Multiplex restriction amplicon sequencing: a novel next‐generation sequencing‐based marker platform for high‐throughput genotyping
Source: Plant Biotechnol J. 2019 Jul 23;18(1):254–65. doi: 10.1111/pbi.13192 (PMC6920337; doi:10.1111/pbi.13192)
Supplement: Supplementary file 9 — Table S6 Sequence of MRASeq primers. [file PBI-18-254-s007.zip › pbi13192-sup-0010-Supinfo.docx]

**Accessing Supplementary Material:**

Once you are logged into ScholarOne, the Home page will be displayed. Please click on the Review tab, where you will find the manuscript listed under "Review and Score". To begin reviewing the manuscript, select “Continue Review” in the “Action” drop down.

You should check the "Files" tab on the Review page as the author may have submitted files that are additional to the main submission, which may not be included in the HTML or PDF but that do require your review. Follow the instructions for reviewers provided on the Instructions tab found on this page. Should you have difficulty in accessing supplementary material then please contact the Editorial Office at [plant-biotechj@wiley.com](mailto:plant-biotechj@wiley.com).
